# Aquaporin-3 mediates ovarian steroid hormone-induced motility of endometrial epithelial cells

**DOI:** 10.1093/humrep/dey290

**Published:** 2018-10-04

**Authors:** Dan Cui, Linlin Sui, Xiao Han, Man Zhang, Zhenzhen Guo, Wanfang Chen, Xinxin Yu, Qiannan Sun, Ming Dong, Tonghui Ma, Ying Kong

**Affiliations:** Core Lab Glycobiol & Glycoengn, College of Basic Medical Sciences, Dalian Medical University, Dalian, Liaoning, China

**Keywords:** AQP3, embryo implantation, ezrin, estrogen, progesterone, endometrial receptivity, epithelial–mesenchymal transition

## Abstract

**STUDY QUESTION:**

How does aquaporin-3 (AQP3) affect endometrial receptivity?

**SUMMARY ANSWER:**

AQP3, which is regulated by the combination and estrogen (E2) and progesterone (P4), induces epithelial–mesenchymal transition (EMT) of endometrial epithelial cells.

**WHAT IS KNOWN ALREADY:**

Embryo implantation is an extremely complex process, and endometrial receptivity is essential for successful embryo implantation. Estrogen and progesterone regulate endometrial receptivity. AQP3, which is regulated by estrogen (E2), increases cell migration and invasion ability by regulating the expression of EMT-related factors and influencing the reorganization of the actin cytoskeleton.

**STUDY DESIGN, SIZE, DURATION:**

This study investigated the pathophysiological significance of AQP3 in human endometrial function during different phases of the menstrual cycle.

**PARTICIPANTS/MATERIALS, SETTING, METHODS:**

AQP3 expression levels during different phases of the menstrual cycle were measured using immunohistochemical assays. In cells of different receptivity (high-receptive RL95-2 cells and low-receptive HEC-1A cells), the expression of AQP3 was measured using western blotting, qRT-PCR and immunofluorescence assays. Activities of AQP3, and its regulation by E2 and P4, were studied through *in-vitro* experiments using RL95-2 cells.

**MAIN RESULTS AND THE ROLE OF CHANCE:**

AQP3 expression in the mid- and late-secretory phases of the human endometrium is significantly higher than in other phases. Since AQP3 expression levels were higher in RL95-2 cells than in HEC-1A cells, mechanisms of AQP3 regulation by E2 and P4 were studied using RL95-2 cells. We provided the first report that P4 up-regulates AQP3 by directly targeting the promoter of the AQP3 gene. The up-regulation of AQP3 expression by a combination of E2 and P4 is significantly higher than that caused by either E2 or P4 alone. Together E2 and P4 promote RL95-2 cell migration and invasion by inducing EMT through AQP3. We also found that AQP3 co-localizes with ezrin and affects the formation of filopodia and lamellipodia during the E2 and P4-induced EMT process but has no effect on the expression of ezrin and F-actin.

**LARGE SCALE DATA:**

N/A.

**LIMITATIONS, REASONS FOR CAUTION:**

It is still unclear whether AQP3 is a main regulator of endometrial receptivity or one of several factors influencing the process.

**WIDER IMPLICATIONS OF THE FINDINGS:**

Further investigation on AQP3 may contribute to a greater understanding of endometrial receptivity.

**STUDY FUNDING/COMPETING INTEREST(S):**

This work was supported by the National Natural Scientific Grants of China (No. 31570798), the Program for Liaoning Excellent Talents in University (LR2017042), the Doctoral Scientific Research Foundation of Liaoning province (201601236), and the Liaoning Provincial Program for Top Discipline of Basic Medical Sciences. There are no conflicts of interest.

## Introduction

The human endometrium is specialized to allow reproductive successful reproduction. In humans, differentiation of the endometrial epithelial cells (EECs) is regulated across the menstrual cycle through changing levels of ovarian steroids. During the menstrual cycle, the endometrium first enters a proliferative phase, and then enters a secretory phase, in which the endometrium becomes receptive to an embryo (Tan *et al.*, 2012; Xie *et al.*, 2014). During embryonal outgrowth, in order to create a space for embryonic penetration, EECs need to apoptosis and migrate away from the implantation site. EEC motility can reconstruct the endometrial epithelial sheet during embryonic invasion. Then, the embryo is covered with epithelial cells and the EEC barrier is restored. Up-regulation of EEC motility is induced by ovarian steroid hormones and implantation-mediated epithelial–mesenchymal transition (EMT). During EMT, the phenomenon known as the ‘cadherin switch’ which is characterized by down-regulation of E-cadherin and up-regulation of N-cadherin, is observed (Uchida *et al.*, 2012). This cadherin switch is associated with actin rearrangement, such as stress fiber formation and decreased cortical actin, resulting in the acceleration of cell motility during EMT.

AQPs are open transport molecules that rapidly transport water and glycerin molecules across a membrane and do not require energy consumption. In mammals, there are 13 subtypes of AQPs (AQP0–AQP12), which show a wide range of distribution in different organs. AQP3 is found in the renal collecting duct, epidermis, conjunctiva and mammary glands, and uterine luminal epithelial cells, among other sites (Mobasheri *et al.*, 2005). Most research on the function of AQP3 has focused on cancer, and few studies regarding the relationship between AQP3 and human reproduction have been published. It has been reported that AQP3 facilitates epidermal cell migration and invasion and lamellipodia formation by transporting water and glycerol (Hara-Chikuma and Verkman, 2008). In breast cancer, increased AQP3 expression affects cell migration and invasion by regulating the expression of EMT-related factors and influencing the reorganization of the actin cytoskeleton (Huang *et al.*, 2014). Other studies have shown that AQP3 knockout in mice leads to defects in urinary-concentrating function (Ma *et al.*, 2000), defects in skin wound healing (Hara-Chikuma and Verkman, 2008) and defects in alimentary tract repair (Thiagarajah *et al.*, 2007).

It has been reported that progesterone combined with estrogen for luteal phase support, compared with progesterone alone, is associated with a higher clinical pregnancy rate in women undergoing IVF (Zhang *et al.*, 2015). Ovarian steroid hormones guide uterine receptivity and successful implantation. An estrogen response element (ERE) has been identified in the promoter region of the AQP1, AQP2 and AQP5 genes (Zou *et al.*, 2013) (Zou *et al.*, 2011). (Jiang *et al.*, 2015). Studies have shown that an ERE in the AQP3 gene mediates estrogen-induced cell migration and invasion in estrogen receptor-positive breast cancer cells (Huang *et al.*, 2014). AQP2 expression in the human endometrium can be regulated by progesterone, although the mechanism remains unclear (He *et al.*, 2006). In addition, it is not clear whether progesterone can directly regulate AQP3 expression.

Ezrin is member of the ezrin–radixin–moesin (ERM) family of membrane–cytoskeletal linkage proteins that facilitate the development of lateral specializations, such as zonae adherens. Ezrin is a major protein in the endometrium and studies have shown that it co-localizes with AQP1 (Fehon *et al.*, 2010; Lecce *et al.*, 2011). Research demonstrates that ezrin associates with cytoskeletal processes underlying many cellular functions including migration, cell division and adhesion (Marion *et al.*, 2011). Ezrin is related to increased invasiveness of tumor cells (Yu *et al.*, 2004; Weng *et al.*, 2005) and poor survival in several cancers (Pang *et al.*, 2004; Yu *et al.*, 2004). Reduced expression of ezrin can inhibit metastasis of cancer cells in several murine and human cancer models. As ezrin is a major protein in the endometrium, and is involved in adhesion and migration, it may play a key role in cytologic changes in endometrial receptivity (Tan *et al.*, 2012).

In the current study, we examined AQP3 expression in the human endometrium throughout the menstrual cycle. We also examined AQP3 expression in E2, P4 and ovarian steroid hormone (E2P4)-treated (Piltonen and Chen *et al.*, 2015; Uchida *et al.*, 2012) human endometrium cells and identified the progesterone receptor in the promoter of the AQP3 gene. We sought to determine the functions of AQP3 in migration, invasion and adhesion and to gain relevant mechanistic insights into these processes. These experiments should contribute to the understanding of the molecular mechanisms of ovarian steroid hormones that are important in remodeling the uterine endometrial receptivity during implantation.

## Materials and Methods

### Tissue collection

All human specimens used in this study were collected from patients with consent during operations in the Department of Obstetrics and Gynecology of The First Affiliated Hospital of Dalian Medical University. Patients were between the ages of 30 and 45 and samples were collected from 2011 to 2014. All procedures were approved by the Institutional Review Board of Dalian Medical University (Liaoning, China). No patients received hormone treatment prior to surgery. The paraffin-fixed pathologic specimens had histopathologic-confirmed diagnoses by in-house experts, and phases as follows: early proliferative, *n* = 9; mid-proliferative, *n* = 9; late proliferative, *n* = 9; early secretory, *n* = 9; mid-secretory, *n* = 9; and late secretory, *n* = 9.

### Cell culture

HEC-1A cells and JAR cells (American Type Culture Collection, Manassas, VA, USA) were grown in McCoy’s 5 A and RPMI 1640 medium. RL95-2 cells were grown in DMEM/F12 (1:1) with 0.005 mg/mL insulin. The three cells lines were all supplemented with 10% FBS, 100 U/ml penicillin and 100 μg/ml streptomycin. When the cells reached 80% confluence, they were subsequently starved of serum for 3 h before treatment with P4 (Sigma, P0130), E2 (Sigma, E2758) or E2 and P4 (concentrations can be seen in figure legends) under serum-free conditions. Cells were harvested for RNA after treatment 48 h and for protein after treatment 72 h.

### Immunohistochemical analysis

Serial sections (4 μm) were prepared from paraffin-embedded tissues. The sections were fixed at 60°C for 3 h, de-paraffinized in xylene and rehydrated in graded alcohol. The slides were microwaved in citrate buffer (20 min) and then incubated in 3% H_2_O_2_ (20 min). After being washed in PBS, sections were blocked with goat serum (37°C, 30 min) before incubation overnight with AQP3 antibody (Abcam, 1:100), with IgG as a negative control and normal human kidney tissue as a positive control. Sections were incubated with biotinylated secondary antibody (40 min, 37°C) and were then incubated with streptavidin–horseradish peroxidase (HRP) (40 min, 37°C). Positive reactions were visualized with a 3,3*N*-diaminobenzidine tetrahydrochloride (DAB)-peroxidase substrate with hematoxylin counterstaining (4 min). Each sample was given a score in which the intensity of the staining (no staining = 0, low staining = 1, medium staining = 2, strong staining = 3) and the percentage of stained cells was calculated. In normal endometrium, the total score was calculated per compartment per sample, as follows: *H*-score= ∑*Pi* (*i* + 1) (1) where *i* is the intensity of staining from 0 (none) to 3 (strong) and *Pi* is the percentage of stained cells for each given *i* (0–100%).

### RNA isolation and real-time PCR

Total RNA was isolated using TRIzol reagent. The cDNA was synthesized using a RNA PCR Kit (AMV), version 3.0. Real-time PCR was performed with an Applied Biosystems Inc. Step One Plus Real-time PCR system according to the recommendations of the manufacturer (ABI, Beijing, China). The relative amount of specific mRNA was normalized to GAPDH and all PCR reactions were run in triplicate. The results were analyzed using the 2^−ΔΔCt^ method. The primers used were as follows: AQP3:5′-AGACAGCCCCTTCAGGATTT-3′ (forward) and 5′-TCCCTTGCCCTGAATAT CTG-3′ (reverse); GAPDH: 5′-GTGAAGGTCG GAGTCAACG-3′ (forward) and 5′-TGAGGTCAA TGA AGGGGTC-3′ (reverse).

### Western blot

Cells were washed in PBS before incubation with Lysis Buffer (Beyotime Biotechnology) on ice for 15 min centrifugation (9000 *g*), and the supernatant was collected. Equal amounts of protein extracts (50 μg) were separated using 12% sodium dodecyl sulfate (SDS)-polyacrylamide gel electrophoresis (PAGE) and transferred to nitrocellulose (NC) filter membranes. The membranes were blocked in 5% non-fat milk in Tris-buffered saline containing 0.1% Tween 20 (TBST) for 3 h and then incubated overnight with anti-AQP3, anti-ezrin, anti-F-actin, anti-E-cadherin or anti-N-cadherin antibodies at 4°C. An anti-β-tubulin antibody was used to confirm equal loading (1:1000). After being washed with TBST, the membrane was incubated with HRP-conjugated anti-rabbit IgG, anti-goat IgG or anti-mouse IgG (1:2000) (45 min, room temperature) and were processed using enhanced chemiluminescence (ECL) and visualized using Bio-Rad Laboratories. Quantification of each band for the target protein was performed using densitometry analysis (Labworks 4.6). The protein band intensity was quantified by the mean ± SEM of three experiments for each group, as determined from densitometry relative to β-tubulin (1:2000). All antibody information is shown in Supplementary Table S2.

### Indirect immunofluorescence staining

RL95-2 and HEC-1A cells grown on coverslips were fixed in 4% paraformaldehyde. Cells were permeabilized using 0.1% Triton X-100 (Beyotime Biotechnology) for 10 min. Non-specific binding sites were blocked by incubation with 2% BSA (room temperature, 1 h). Samples were then incubated with AQP3 antibody (1:100, Abcam) overnight at 4°C. After being washed three times with PBS, slides were incubated with FITC-conjugated goat anti-rabbit IgG H&L (1:100) for 1 h at 37°C (Invitrogen, Carlsbad, CA). Filamentous actin (F-actin) was detected using FITC-conjugated phalloidin (Sigma, St Louis, Missouri) diluted in phosphate buffer (50 μg/ml). Slides were incubated with 4′,6-diamidino-2-phenylindole (DAPI). Specimens were mounted in PBS containing 90% glycerol and 1.0% P-phenylenediamine. Slides were imaged using an Olympus BX51 immunofluorescence microscope.

RL95-2 cells were incubated with a 1:100 dilution of primary AQP3 (Santa Cruz Biotechnology, Santa Cruz, CA) or ezrin (Cell Signaling Technology) antibody at 4°C overnight. The methods of subsequent experiment are the same as above. Specimens were monitored under laser confocal microscopy with a Leica microscope. (All antibody information is shown in Supplementary Table S2).

### Bioinformation and chromatin immunoprecipitation analyses

The Regulatory Sequence Analysis Tools (RSAT) web server was used to analyze the promoter sequence of AQP3 to find six high-scoring sequences. RL95-2 cells were treated with 10 μM P4 for 6 h and cross-linked with 1% formaldehyde and homogenized in PBS containing 0.125 M glycine. The DNA was sonicated (50 W for 15 s, six times and interference by 30 s). These sonicated samples were precleared with protein A/G Sepharose for 1 h at 4°C and then incubated with either 10 μg progesterone receptor A/B antibody (D8Q2J; Cell Signaling Technology) or 10 μg anti-RNA polymerase II antibody (Cell Signaling Technology) at 4°C overnight. The samples were precipitated with protein A/G Sepharose, washed five times with RIPA buffer, and then recovered by incubation in elution buffer (0.1 M sodium bicarbonate and 1% SDS). Cross-linking was reversed by incubation for 12 h at 65°C, followed by incubation with proteinase K for 4 h at 45°C. Samples were extracted with phenol/chloroform and ethanol precipitated. In general, one-tenth of the precipitated DNA was used in the PCR amplification reaction. PCR was carried out in 10 μl reaction mixtures containing 2 μl of 5 × PCR La-buffer (with Mg^2+^), 2 μl of four dNTP (each 2.5 mM), 0.25 μl of forward and reverse primers (10 M), and 0.1 μl of La-Taq (TAKARA, Dalian, China). The PCR products were analyzed using agarose electrophoresis. As a positive control, all DNA fragments were precipitated with RNA polymerase II antibody. As a negative control, the same experiments were performed with IgG. Primer sequences used to amplify the precipitated DNA are listed in Supplementary Table S1.

### Plasmid construction and luciferase reporter assay

The RSAT web server was used to analyze the promoter sequence of AQP3 to find four high-scoring sequences. DNA fragments encoding human AQP3 promoters were synthesized by Gene Pharma (Suzhou, China), digested with XhoI and BamII restriction enzymes, and ligated into pGL3-basic (Gene Pharma) to construct the AQP3 promoter-luciferase reporter systems. RL95-2 cells were transfected with 200 ng of DNA fragments encoding human AQP3 promoters and 200 ng of pRL-TK reporter plasmid (cDNA encoding Renilla luciferase) according to the manufacturer’s instructions. RL95-2 cells were treated with 10^−5^ M P4 for an additional 24 h. Luciferase activities in cell lysates were measured using the dual-luciferase reporter assay system (Promega, E1960) according to the manufacturer’s instructions. Luciferase values were normalized to the Renilla luciferase activity. We had constructed seven plasmids: S1, S2, S3, S4, S5, S6 and S1 (D); further information is shown in Supplementary Table S1.

### Short Interfering RNA knockdown and vector-mediated overexpression studies

For knockdown experiments, siRNA targeting the AQP3 gene (50 nmol/well, 200 nmol/well) and siRNA negative control were purchased from Gene Pharma (Suzhou, China). For overexpression experiments, an AQP3 overexpression vector (200 nmol/well, 100 nmol/well) was purchased from Gene Pharma (Suzhou, China). Cell transfection was conducted using Lipofectamine 2000 (Invitrogen, Carlsbad, CA, USA).

### Wound healing assay

RL95-2 and HEC-1A cells were seeded in 6-well plates under serum-free conditions. After pre-treatment, the monolayer cells were then scratched with a standard 10 μl pipette tip and washed with PBS. Cells were cultured in medium supplemented with or without E2 and P4 (10 nM 17β-estradiol + 1 μM progesterone) for 24 h. Wound healing ability was quantified by measuring the migratory distance of cells.

### Transwell invasion assay

A permeable filter of a transwell system (Corning Incorporated, Midland, MI, USA) was used. The inside compartment of the transwell inserts was coated with Matrigel (BD Biosciences, Bedford, MA, USA) (37°C, 3 h) and then blocked with 1% PBS for 30 min. After pre-treatment (AQP3 knockdown or overexpression), RL95-2 and HEC-1A cells (1 × 10^5^/well) were loaded in the upper chamber and incubated with or without E2P4 for 32 h. Cell migration to the other side of the membrane was induced by 20% FBS-containing medium in the lower chamber. Cells were fixed in methanol for 30 min and stained with 0.5% crystal violet for 20 min. After the cells were removed, images were taken and counted using Image J software (National Institutes of Health, Bethesda, MD, USA).

### Adhesion of embryonic JAR cells to RL95-2 and HEC-1A cell monolayers

Percentage adhesion of JAR cells to the endometrial cells was calculated as previously described. In brief, RL95-2 or HEC-1A cells were seeded in 96-well plates to form a confluent monolayer. Differently treated JAR cells were stained with CellTracker™ Green CMFDA (Life Technologies, USA) 1 h before the adhesion assay. The cells were gently seeded onto RL95-2 or HEC-1A cell monolayers in JAR culture medium and under serum-free conditions. After 1 h washing with PBS, the attached cells were detected by using a multimode plate reader (PerkinElmer, USA) and photographed using a fluorescent phase contrast microscope (Olympus, Japan).

### Co-immunoprecipitation

For co-immunoprecipitation, RL95-2 cells were lysed in a solution including 25 mM HEPES, 100 mM NaCl, 1 mM EDTA, 0.5 mM MgCl_2_, 10 mM NaF, 1 mM phenylmethylsulfonyl fluoride, 1 mM Na orthovanadate, 1 mM aprotinin, 1 mM leupeptin, 10% glycerol and 1% Nonidet P-40 (pH 7.5). Lysates were placed on ice for 30 min and then centrifuged for 20 min at 14 000 *g*. The supernatant was incubated with anti-AQP3 antibody (Santa Cruz Biotechnology, Santa Cruz, CA) or anti-ezrin antibody at 4°C overnight and then precipitated with protein A/G agarose beads (Santa Cruz Biotechnology, Santa Cruz, CA). As a negative control, the same experiments were performed with IgG. The precipitates were washed three times in lysis buffer and subjected to western blot analysis.

### Statistical analysis

All data were normally distributed and expressed as mean ± SD. The independent-samples *t*-test was used to evaluate the statistical significance between two groups. One-way ANOVA was used to evaluate the statistical significance of the difference between more than two groups. The SPSS version 13.0 for Windows was used for the statistical analysis. *P* < 0.05 was considered statistically significant.

## Results

### AQP3 expression in the human menstrual endometrium

**Figure 1:**
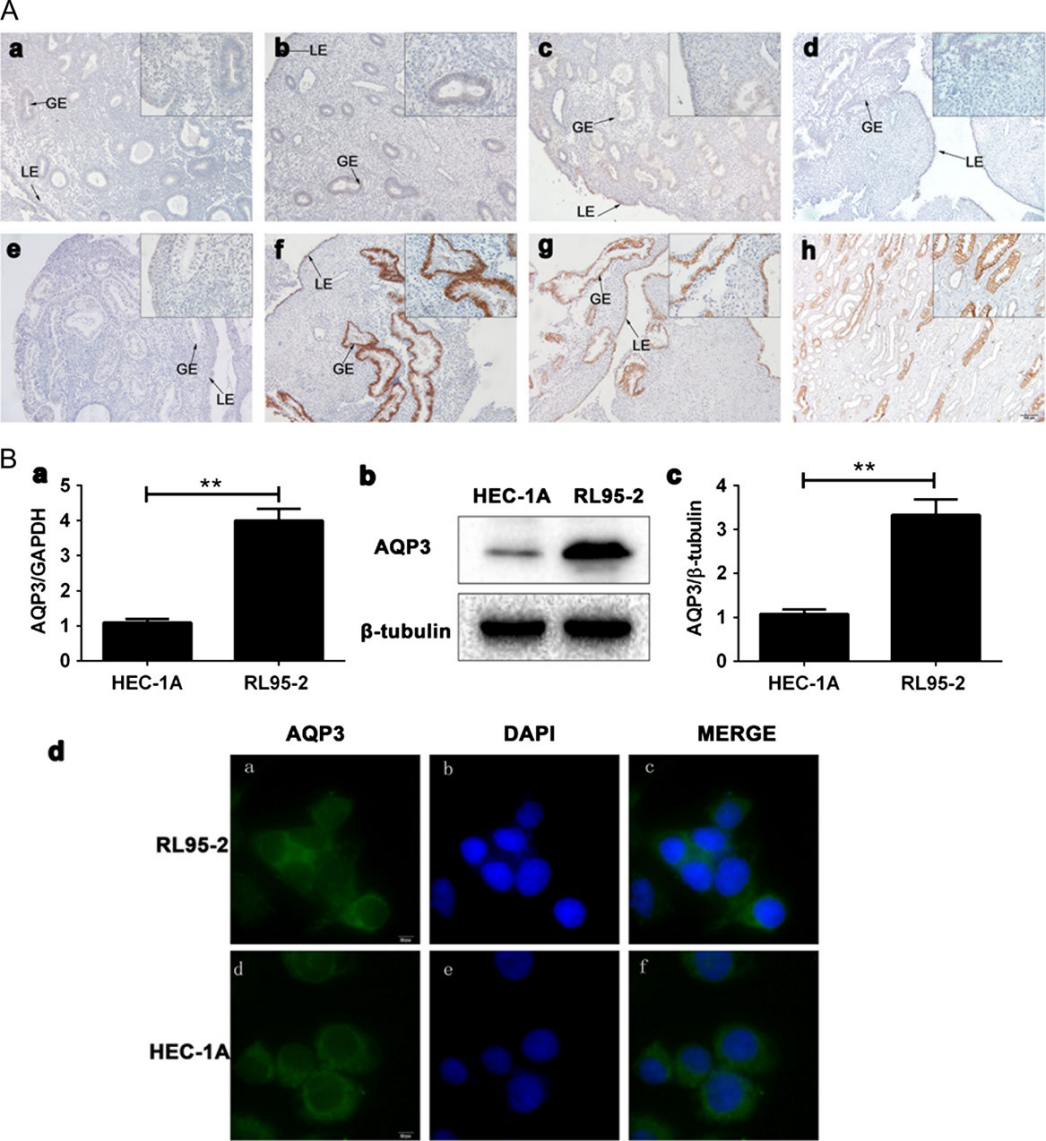
AQP3 expression in the human endometrium. (**A**) Paraffin-embedded endometrial tissues were analyzed by immunohistochemistry at stages of the various menstrual cycle. AQP3 expression in the early (a), mid- (b) and late (c) proliferative phases. The mid-secretory phase (isotype IgG, d) was used as negative control. AQP3 expression in early (e), mid- (f) and late (g) secretory phases. Kidney was used as positive control (h). LE, lumina epithelium. GE, glandular epithelium. (**B**) AQP3 expression in RL95-2 and HEC-1A cells. AQP3 mRNA expression in RL95-2 and HEC-1A cells analyzed by real-time PCR (a). AQP3 protein expression analyzed by Western blot in RL95-2 and HEC-1A cells (b, c). AQP3 protein expression detected by indirect immunofluorescence staining in RL95-2 (a–c) and HEC-1A (d–f) cells. Experiments were repeated five times. The data are presented as the means ± SD, *P < 0.05 and **P < 0.01 (Student’s *t*-test). All images are magnified ×400. Bar = 20 μm. GAPDH, glyceraldehyde 3-phosphate dehydrogenase.

To explore the pathophysiological significance of AQP3 in human endometrial function, we collected human endometrial biopsies during different phases of the menstrual cycle from 54 normally cycling volunteers. Immunohistochemistry results demonstrated that AQP3 was minimally expressed in the early proliferative (Fig. 1Aa), mid-proliferative (Fig. 1Ab), late-proliferative (Fig. 1Ac) and early-secretory (Fig. 1Ae) phases. In the mid- (Fig. 1Af) and late-secretory (Fig. 1Ag) phases, AQP3 was expressed strongly in both the luminal and glandular epithelia (Fig. 1A, LE, GE) and peaked in the mid-secretory phase (Table I) (*P* < 0.01). However, AQP3 expression was not observed in stromal cells in any phase of the menstrual cycle. To assess whether AQP3 expression associates with endometrial epithelium receptivity, expression profiles were examined in RL95-2 and HEC-1A cells by q-PCR (Fig. 1Ba), western blotting (Fig. 1Bb,c) and indirect immunofluorescence staining (Fig. 1Bd). AQP3 mRNA and protein expression levels were higher in high-receptive RL95-2 cells than in low-receptive HEC-1A cells (*P* < 0.01).

**Table I:** Expression of AQP3 protein image analysis in human endometrial epithelium of different phases.

	EP	MP	LP	ES	MS	LS
*H*-score*	1.12 ± 0.06^a^	1.21 ± 0.09^b^	1.15 ± 0.08^c^	1.12 ± 0.12^d^	3.88 ± 0.19^e^	3.01 ± 0.12 ^f^

EP, early proliferative phase; MP, mid-proliferative phase LP, late-proliferative phase; ES, early-secretory phase; MS, mid-secretory phase; LE, late-secretory phase. Results were expressed as the mean+ SEM of *H*-score. ^e-a, e-b, e-c, e-d^Significant difference (*P* < 0.01); ^f-a, f-b, f-c, f-d^significant difference (*P* < 0.01); ^e-f^significant difference (*P* < 0.05). *H*-score=ΣPi(*i *+ 1), where *i* represents 0 (none) to 3 (very strong) and Pi=percentage of cells for each intensity (100%).

### The effect of estrogen and progesterone on AQP3 in RL95-2 and HEC-1A cells

**Figure 2:**
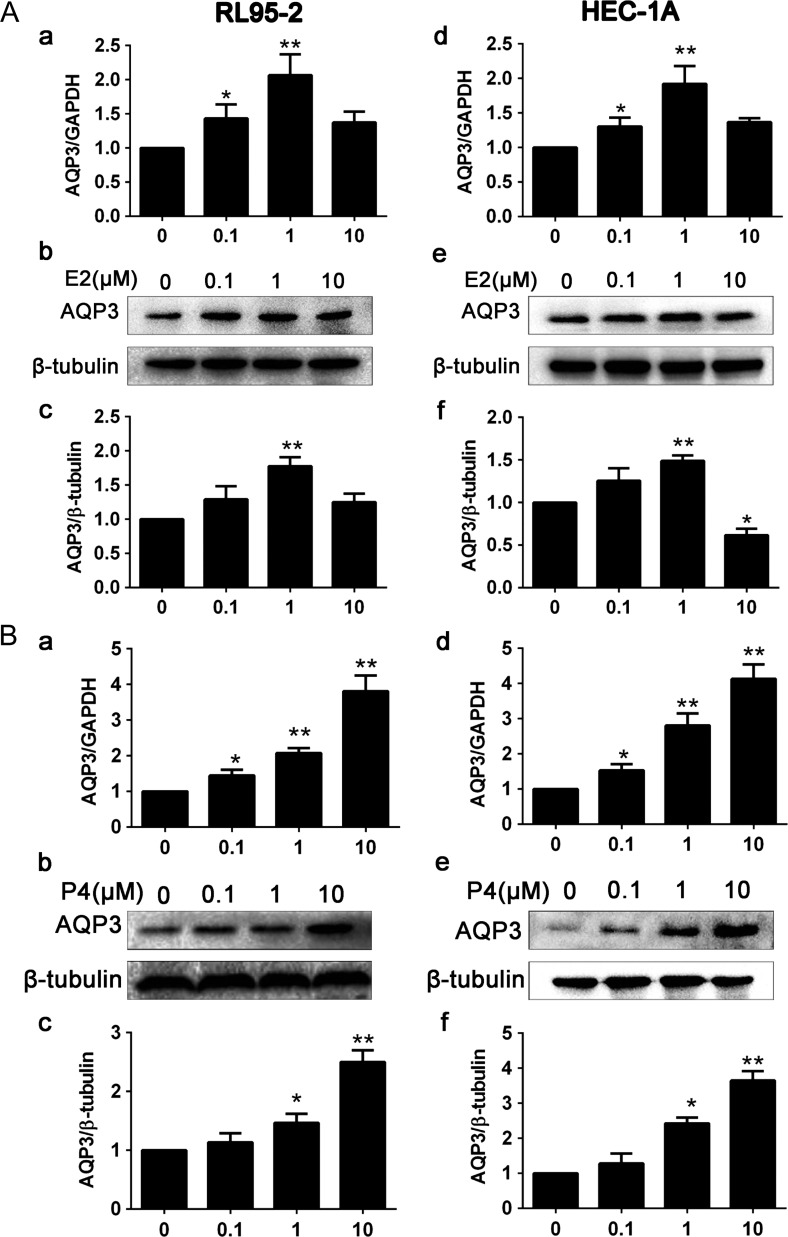
Effects of progesterone and estrogen on AQP3 expression in RL95-2 and HEC-1A cells. Cells were treated with different concentrations of estrogen. (**A**) Real-time PCR analysis of AQP3 expression in RL95-2 cells (a) and in HEC-1A cells (d). Western blot detection of AQP3 expression in RL95-2 cells (b, c) and in HEC-1A cells (e, f). (**B**) Cells were treated with different concentrations of progesterone. AQP3 expression analysis by real-time PCR in RL95-2 cells (a) and in HEC-1A cells (d). Western blot analysis of AQP3 expression in RL95-2 cells (b, c) and HEC-1A cells (e, f). Experiments were repeated four times The data are presented as the means ± SD, *P < 0.05 and **P < 0.01 (Student’s *t*-test). The concentrations of progesterone and estrogen used were 0.1, 1 and 10 μM under serum-free conditions.

The ovarian steroids hormone, estrogen and progesterone are the principal hormones that direct uterine receptivity and are very important during the menstrual cycle. To determine whether estrogen regulates AQP3 expression in RL95-2 cells and HEC-1A cells, we treated the two cell lines with estradiol (E2). We found that E2-induced up-regulation of AQP3 mRNA and protein expression in RL95-2 cells (Fig. 2Aa,b,c) and HEC-1A cells (Fig. 2Ad,e,f) in a dose-dependent manner, at concentrations ranging from 0.1 to 1 μM (*P* < 0.05). As the estrogen concentration increased from 0.1 to 10 μM, the expression of AQP3 increased accordingly and peaked at 1 μM. To determine whether progesterone regulates AQP3 expression in RL95-2 cells and HEC-1A cells, we treated the two cell lines with progesterone (P4). We found that AQP3 expression at both the mRNA (Fig. 2Ba,d) and protein (Fig. 2Bb,c,e,f) levels was stimulated by P4 in a dose-dependent manner at concentrations ranging from 0.1 to 10 μM (*P* < 0.01).

### Progesterone up-regulates AQP3 in RL95-2 cells via a PRE in the AQP3 gene

**Figure 3:**
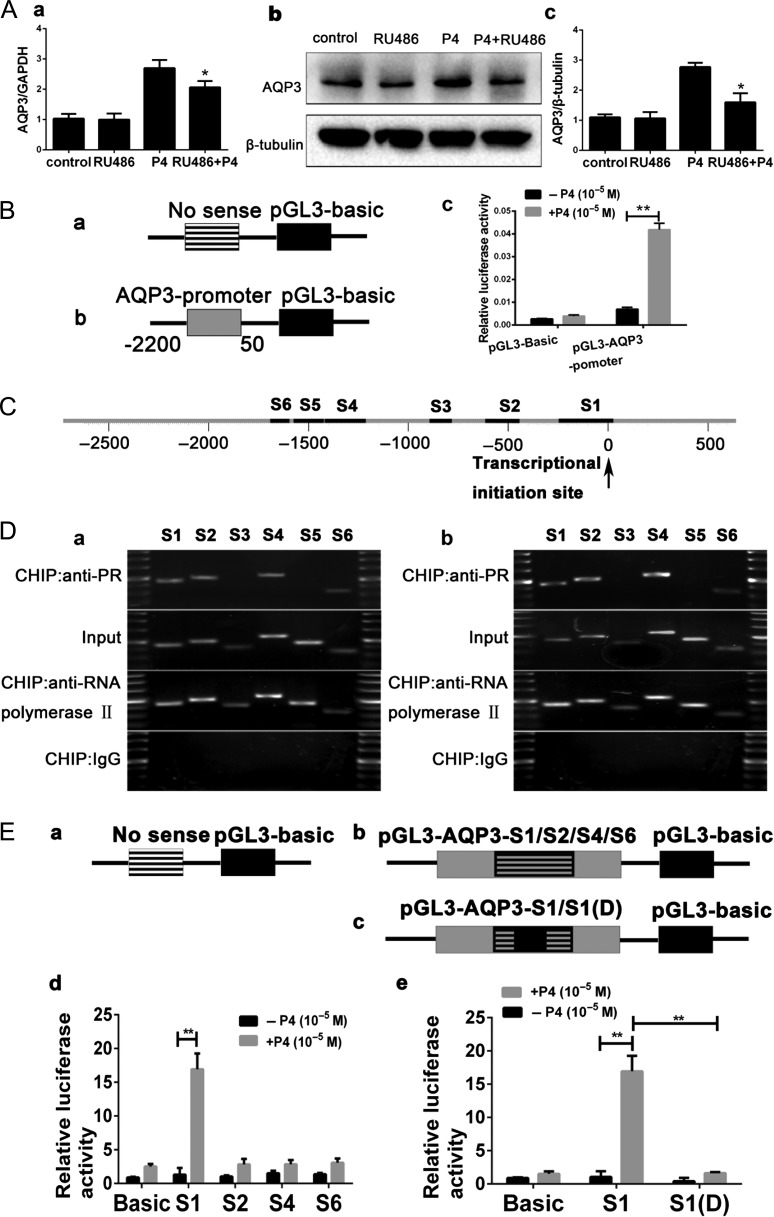
Identification of the functional PRE in the promoter of AQP3 gene. (**A**) PR antagonist RU486 can reduce the expression of AQP3 mRNA (a) and protein (b, c), which induced by P4. (**B**) Schematic diagram of AQP3 promoter-driven luciferase reporter constructs. PGL3-Basic (negative control) (a) and PGL3-AQP3-promoter containing the sequences of the AQP3 promoter (50 ~−2200 bp) (b). PGL3-AQP3-promoter could be activated by P4, and mutated pGL3- Basic could not be activated by P4, as detected by luciferase activities of the report systems (c). (**C**) Schematic depiction of the six potential AQP3 promoter sequences (S1–S6, black rectangles) that might contain putative PREs. (**D**) ChIP analysis included positive control (anti-RNA polymerase II), negative control (normal mouse IgG), PR antibody and input groups. Four sequences (S1, S2, S4 and S6) were pulled down more by anti-PR antibody in the presence of P4 (b) than in the absence of P4 (a). After sequencing, S1, S2, S4 and S5 were included (not shown in the figure). (**E**) Schematic diagram of AQP3 promoter-driven luciferase reporter constructs: PGL3-Basic (negative control) (a) PGL3-AQP3-S1, pGL3-AQP3-S2, pGL3-AQP3-S3 and pGL3-AQP3-S4 contained S1, S2, S3 and S4, respectively (b) pGL3-AQP3 -S1 (D) contained a deleted S1(c). Luciferase activities of the report systems showed that pGL3-AQP3-S1 could be activated by P4 (d), P4 had no effect on the mutated AQP3-luc-S1 (D) (e). Experiments were repeated four times. The data are presented as mean±SD, **P < 0.01 (Student *t*-test) (progesterone concentration: 10 μM).

To determine whether the P4 receptor (PR) mediates the effects of progesterone, RL95-2 cells were pretreated with RU486, a Progestin receptor antagonist (Murad and Gilman, 1975; Fiala and Danielsson, 2006), for 1 h before P4 treatment. The P4-mediated increases in AQP3 mRNA (Fig. 3Aa) and protein (Fig. 3Ab,c) levels were decreased by 10 μM RU486 treatment, indicating that PR is involved. We further investigated the involvement of PR in P4-induced nuclear translocation in the RL95-2 cells using a luciferase reporter system (AQP3-promoter-luc). A fragment of the AQP3 promoter (50 ~ −2200 bp) was ligated with the luciferase expression vector pGL3 and was used to indicate promoter function (Fig. 3Ba,b). Further investigation showed that 10 μΜ P4 significantly enhanced the luciferase activity in the AQP3-promoter-luciferase system (Fig. 3Bc). This result suggests that AQP3 may be a direct target gene of progesterone.

We performed bioinformatic analysis and conventional ChIP to confirm the fragments of AQP3 promoter. Bioinformatics analysis show there are six high-scoring sequences which we named S1, S2, S3, S4, S5 and S6 (Fig. 3C, Supplementary Table S1). ChIP analysis verified that the PRE is located in the S1, S2, S4 and S6 fragments of the AQP3 promoter region (Fig. 3Da,b). To confirm this result, we constructed four plasmids (pGL3-AQP3-S1, pGL3-AQP3-S2, pGL3-AQP3-S4 and pGL3-AQP3-S6) for a luciferase reporter assay (Fig. 3Ea,b). P4 induced significant increases in the AQP3 promoter activity in RL95-2 cells pre-transfected with pGL3-AQP3-S1 (Fig. 3Ed). Furthermore, the results were confirmed by constructing pGL3-AQP3-S1(D) plasmids (with the ‘CTGCAGGGCGGGCGGGGCCCGTGTCTCCAGCGCTCCTA TAAAGGGAGCCA’ sequence deleted; all sequence information can be seen in Supplementary Table S1) (Fig. 3Ec,e). These results indicate that the P4 mediates the regulation of AQP3 expression by directly targeting the promoter of the AQP3 gene in human endometrial cells.

### AQP3 is required for endometrial cell migration and invasion but not for adhesion

**Figure 4:**
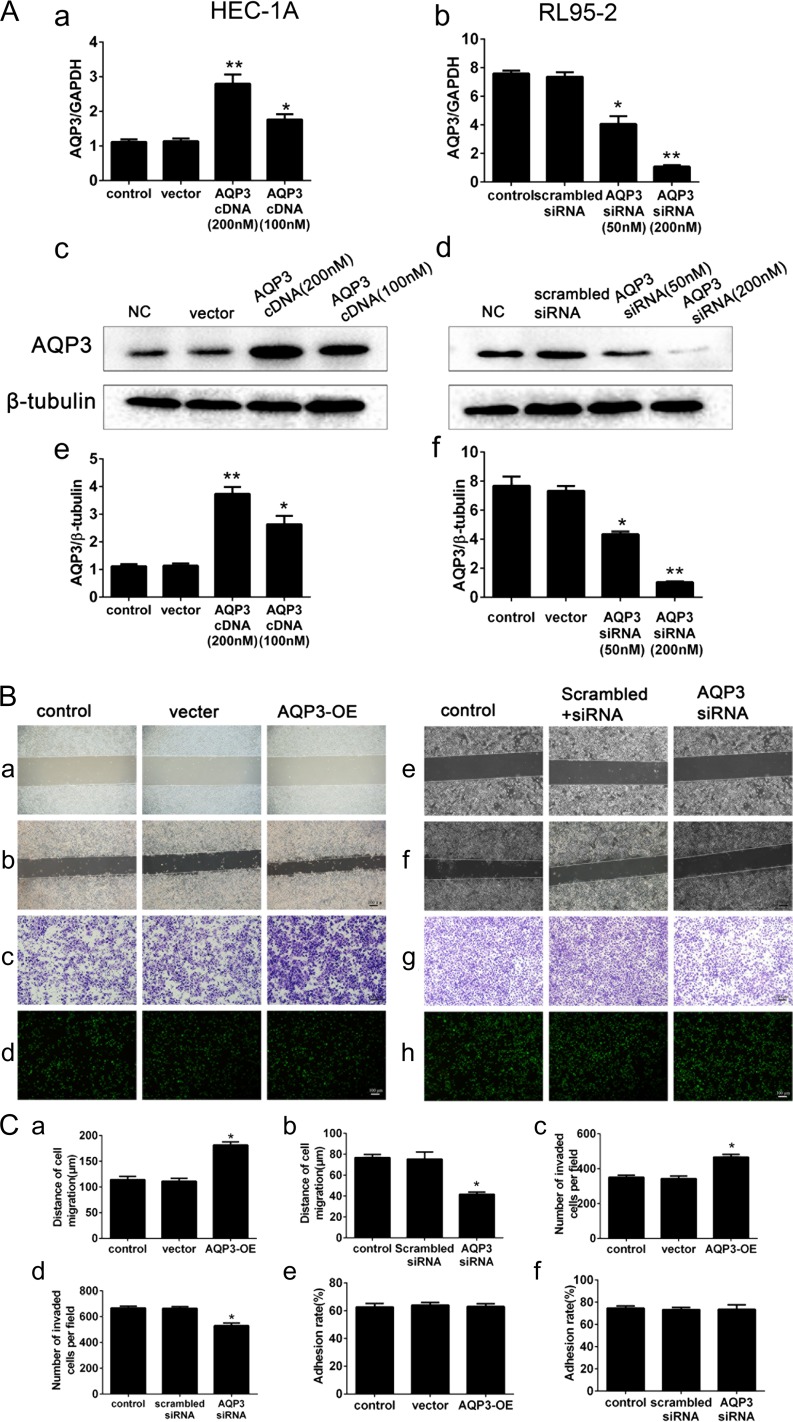
Regulation of AQP3 expression by transfection of AQP3 overexpression plasmids and the function of AQP3 in RL95-2 and HEC-1A cells. (**A**) Real-time PCR of AQP3 expression at the gene level in HEC-1A cells after transfection with over-AQP3 (a). Real-time PCR of AQP3 expression in RL95-2 cells after transfection with AQP3 siRNA (b). AQP3 expression at the protein level, determined by western blotting after transfection with over-AQP3 (AQP3-OE) in HEC-1A cells (c, e) and in RL95-2 cells (d, f). (**B**) AQP3 increased cell migration (HEC-1A, B, a (0 h), b (48 h), **C**, a; RL95-2, B, e (0 h), f (48 h), C, b) and cell invasion (B, c, g; C, c, d). Adhesion assay of JAR cells on HEC-1A (Bd) and RL95-2 (Bh) cell monolayers. Cells were stained with CellTracker™ Green CMFDA 1 h before the adhesion assay and were photographed with a fluorescence microscope. Adhesion rate was measured using a multimode plate reader (C, e, f). The experiments were repeated six times. All images are magnified ×100. Bar = 100 μm. The data are presented as the means ± SD, *P < 0.05 and **P < 0.01 (Student’s *t*-test).

To explore the role of AQP3 in endometrial epithelium receptivity, we transfected siRNAs targeting the AQP3 gene or an AQP3 overexpression plasmid (over-AQP3) into RL95-2 (high AQP3 expressing cells) and HEC-1A (low AQP3 expressing cells) cells and analyzed migration, invasion and adhesion. AQP3 expression was much higher in HEC-1A cells transfected with over-AQP3 than in the control group. Transfection with the over-AQP3 plasmid strongly increased the AQP3 mRNA (Fig. 4Aa) and protein (Fig. 4Ac,e) expression levels in HEC-1A cells (*P* < 0.01). HEC-1A cells transfected with 200 nM AQP3 cDNA showed increased cell migration (Fig. 4Ba(−0 h),b(−48 h) and Ca) and invasion (Fig. 4Bc and Cc), as compared to the vector-transfected and non-transfected control cells. However, *in-vitro* experimental studies using trophoblastic spheroids made from JAR cells line as the embryo surrogate (Evron *et al.*, 2011; Zhang *et al.*, 2011; Li *et al.*, 2017) found that up-regulation of AQP3 had no effect on JAR cells adhesion to HEC-1A cells (Fig. 4Bd and Ce). In contrast, AQP3 mRNA expression in AQP3 siRNA-transfected RL95-2 cells was dramatically lower than in the control group, as determined by RT-PCR (Fig. 4Ab). Similar results were obtained for protein expression as measured by western blotting (Fig. 4Ad,f). These results show that AQP3 siRNA effectively suppresses AQP3 expression in RL95 cells (*P* < 0.01). AQP3 siRNA-transfected (200 nmol/l) RL95-2 cells exhibited significantly decreased cell migration (Fig. 4Be(−0 h),f(−48 h) and Cb) and invasion (Fig. 4Bg and Cd) as compared with the scrambled siRNA-transfected and non-transfected control cells (*P* < 0.05). However, AQP3 knockdown had no effect on JAR cell adhesion to RL95-2 cells (Fig. 4Bh and Cf).

### E2 and P4 promotes RL95-2 cell migration and invasion through AQP3

**Figure 5:**
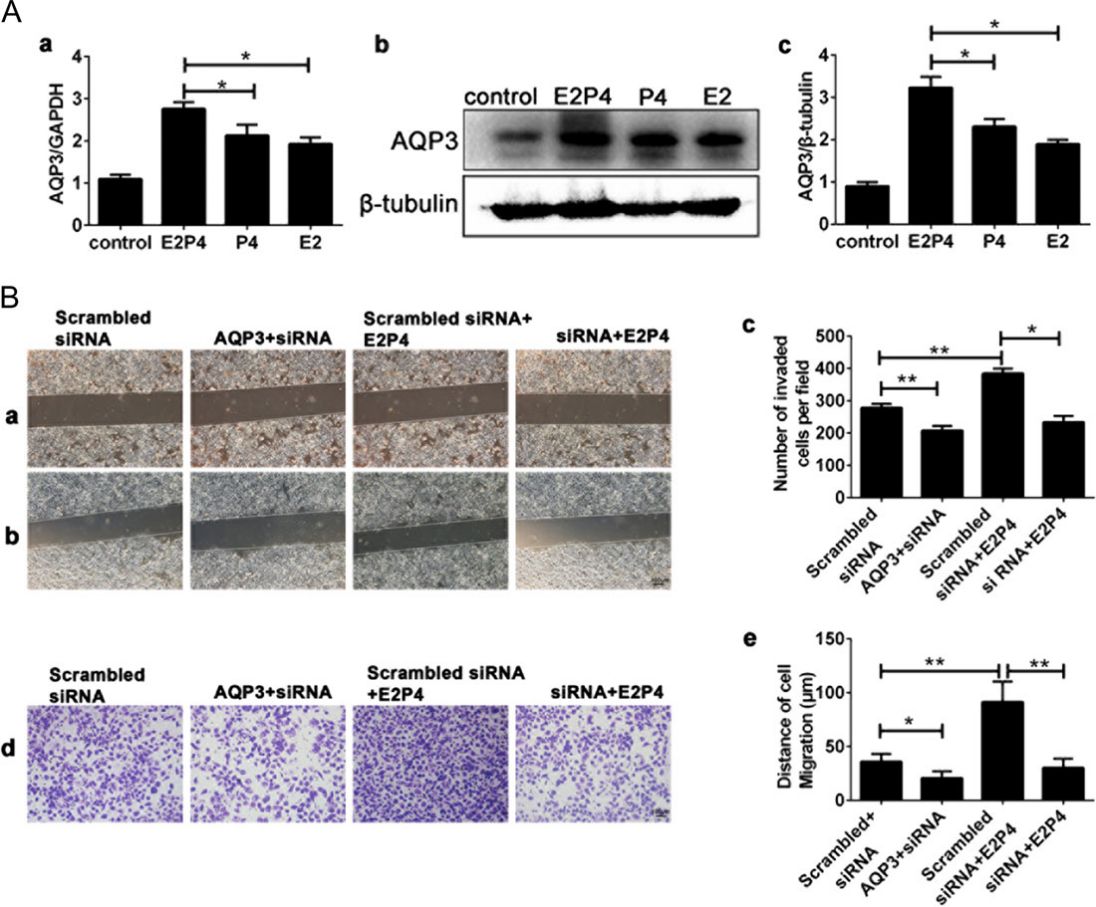
Effects of the ovarian steroid hormones (17β-estradiol and progesterone, E2P4) on AQP3 in expression RL95-2 cells. (**A**) Real-time PCR analysis of AQP3 mRNA in RL95-2 cells (a). Western blot detection of AQP3 protein in RL95-2 cells (b, c). GAPDH, glyceraldehyde 3-phosphate dehydrogenase. Each experiment was repeated seven times. (**B**) Knockdown of AQP3 reduced E2P4-induced cell migration (B, a(0 h), b(48 h), c) and invasion (Bd, e). All images are magnified ×100. Bar = 100 μm. The data are presented as the means ± SD, *P < 0.05 and **P < 0.01 (Student’s *t*-test). Experiments were repeated six times. Estradiol concentration: 10 nM; progesterone concentration: 1 μM; E2P4 concentration: 10 nM estradiol and 1 μM progesterone.

E2 and P4 treatment up-regulated AQP3 expression at both the mRNA (Fig. 5Aa) and protein (Fig. 5Ab,c) levels more significantly than either estrogen or progesterone alone. E2 and P4 promoted migration (Fig. 5Ba-(0 h),b-(48 h),Bc) and invasion (Fig. 5Bd,e) of RL95-2 cells through regulating AQP3 expression.

### AQP3 co-localizes with ezrin and knockdown of AQP3 has no effect on the expression of ezrin in RL95-2 cells

**Figure 6:**
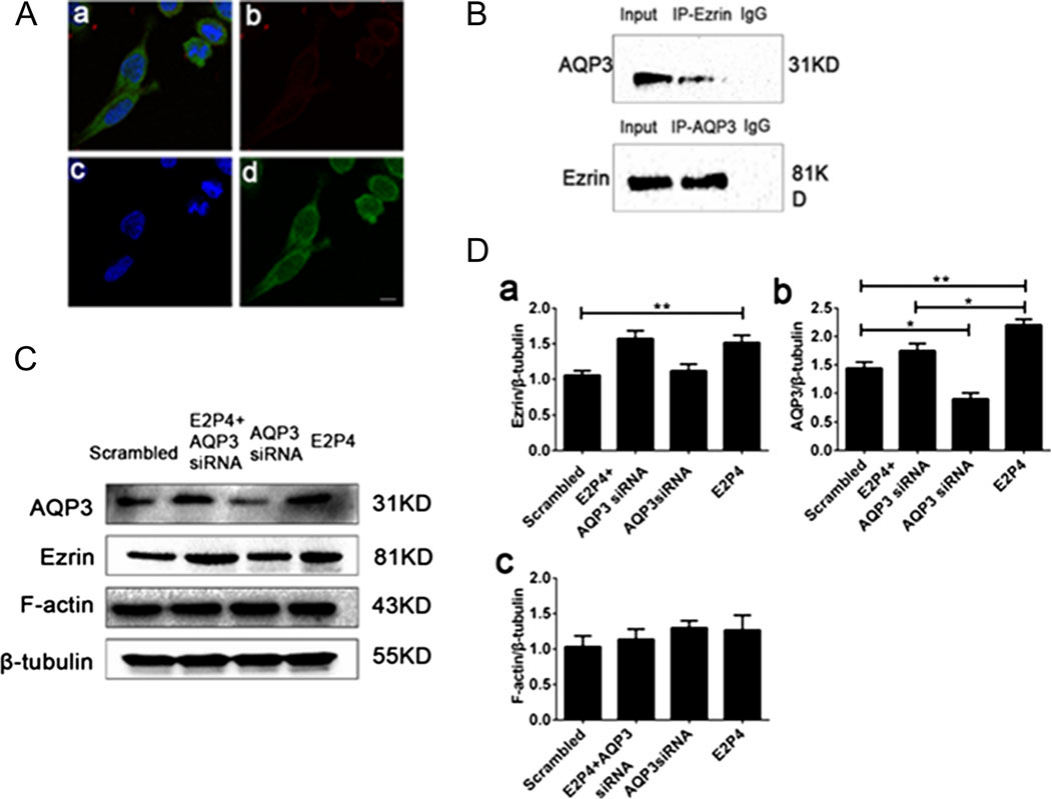
(**A**) Co-immunolocalization of AQP3 and ezrin. Ezrin and AQP3 are visualized in green (d) and red (b), respectively; nuclear DNA (c) is stained blue with DAPI; merged image of b, c, d (a). (**B**) Co-immunoprecipitation of ezrin and AQP3 in RL95-2 cells. (**C**) Representative western blot of ezrin and F-actin expression after treatment of RL95-2 cells with scrambled siRNA, AQP3 siRNA+E2P4, AQP3 siRNA, and AQP3 siRNA+E2P4, respectively. (**D**) Summary data of the western blot analyses (a, b, c). Experiments were repeated five times. Data are presented as the means ± SD, *P < 0.05 and **P < 0.01 (Student’s *t*-test). The images are magnified ×630. Bar = 10 μm. Abbreviation: IP, immunoprecipitation. E2P4 concentration: 10 nM estradiol and 1 μM progesterone.

To explore the role of AQP3 in EMT of human endometrial high-receptive RL95-2 cells in human embryo implantation induced by E2P4, we utilized AQP3-specific siRNA and E2 and P4. Co-immunolocalization showed that AQP3 co-localized with ezrin in RL95-2 cells (Fig. 6A). This was further confirmed by co-immunoprecipitation and AQP3 was detected in the ezrin immunoprecipitate in the converse experiment (Fig. 6B). Furthermore, neither ezrin nor AQP3 was detected in the corresponding IgG control precipitates. It has been reported that ezrin co-localizes with F-actin (Ferreira *et al.*, 2017). To elucidate the relationship between AQP3 and ezrin, we examined ezrin expression in RL95-2 cells treated with and without siRNA targeting AQP3 (Fig. 6C and Da,b). E2 and P4 significantly increased ezrin protein expression. Treatment of RL95-2 cells with AQP3 siRNA did not inhibit the increased ezrin expression induced by E2 and P4 treatment. However, neither E2 and P4 nor AQP3 siRNA altered F-actin expression (Fig. 6C and Dc). These results suggest that AQP3 expression has no effect on the expression of ezrin or F-actin during the EMT process.

### E2 and P4 up-regulates AQP3-influenced reorganization of actin cytoskeleton, ezrin organization and the expression of EMT-related factors in RL95-2 cells

**Figure 7:**
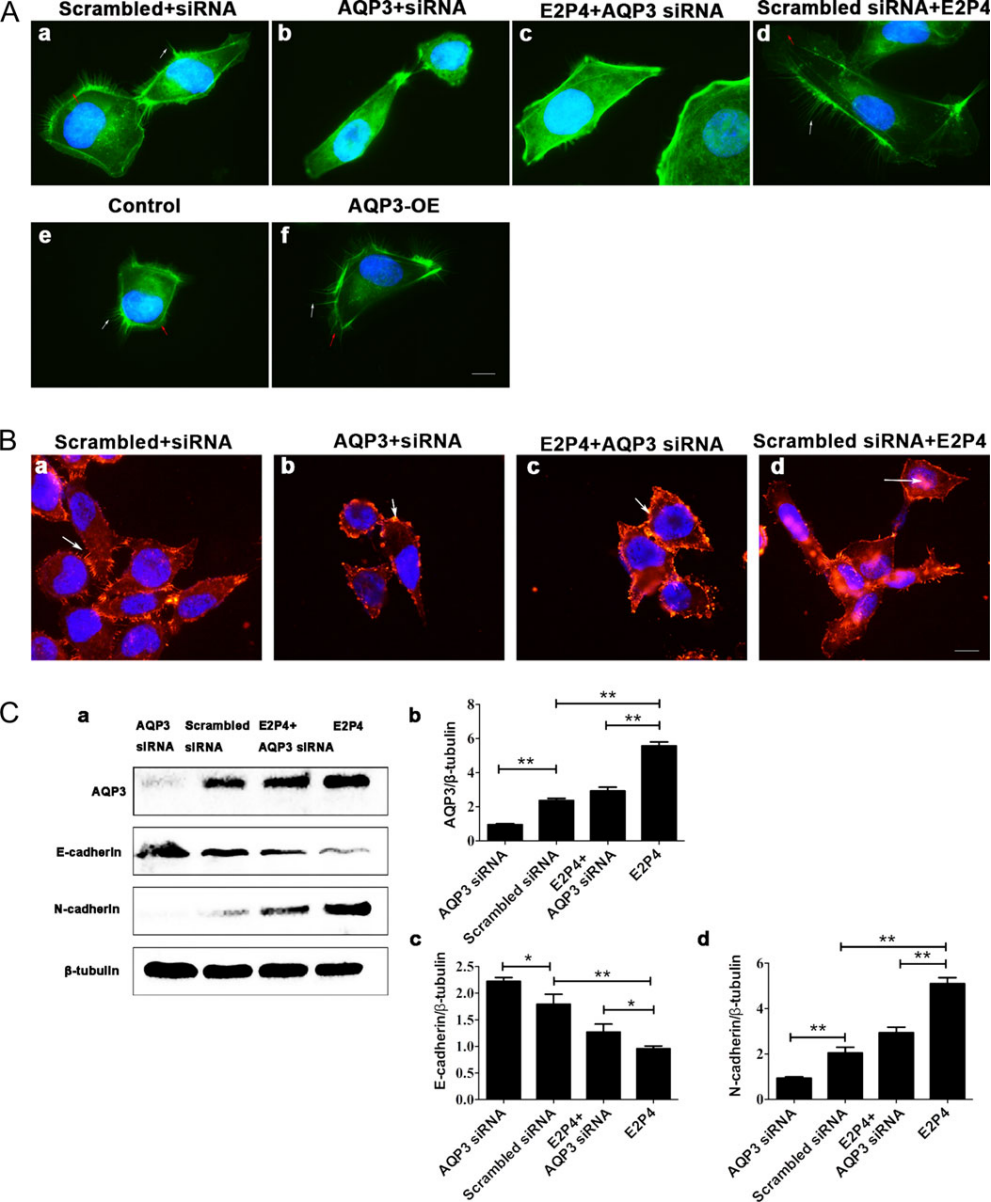
AQP3 influenced reorganization of the actin cytoskeleton and ezrin. (**A**) Organization of the actin cytoskeleton in RL95-2 cells treated with or without AQP3 siRNA, respectively (a, b). E2P4 induced the reorganization of the actin cytoskeleton by promoting filopodia formation and stress fiber rearrangement (d). Treating RL95-2 cells with AQP3 siRNA (c) significantly reduced E2P4-regulated reorganization of the actin cytoskeleton. AQP3 overexpression influenced reorganization of the actin cytoskeleton (f). The images are magnified ×1000. Bar = 5 μm. White arrows indicate filopodia; red arrows indicate lamellipodia. Experiments were repeated four times. (**B**) Ezrin organization in RL95-2 cells treated with or without AQP3 siRNA, respectively (a, b). E2P4 induced ezrin reorganization by promoting ezrin expression (d). Treating RL95-2 cells with AQP3 siRNA (c) significantly reduced E2P4-regulated ezrin reorganization but not its expression. The images are magnified ×630. Bar = 15 μm. White arrows indicate ezrin. Experiments were repeated four times. (**C**) AQP3 influenced the expression of EMT-related factors. Knockdown of AQP3 in RL95-2 cells inhibited the E2P4-induced EMT (a–d). The data are presented as the means ± SD, *P < 0.05 and **P < 0.01 (Student’s *t*-test). Experiments were repeated six times. E2P4 concentration: 10 nM estradiol and 1 μM progesterone.

To explore whether AQP3 affects the organization of the actin cytoskeleton, Filamentous actin (F-actin) was detected using fluorescein isothiocyanate-conjugated phalloidin. Using microscopy, we found that AQP3 overexpression (Fig. 7Ae,f) (versus the control) and E2 and P4 (Fig. 7Aa,c) (versus the scrambled+siRNA group and E2P4+AQP3 siRNA) induced the formation of filopodia. In contrast, in the AQP3 siRNA-treated groups (versus the scrambled+siRNA group), the F-actin loops around the cell nuclei and the formation of filopodia was reduced (Fig. 7Aa,b,c,d). We also found that AQP3 had an effect on ezrin organization. In normal RL95-2 cells, ezrin was localized in the cortical region beneath the membrane and was enriched in the cellular pseudopod evenly (Fig. 7Ba). Treatment with E2P4 promoted the expression of Ezrin, and redistributed ezrin redistributed inside the cytoplasm (Fig. 7Bd). By inhibiting the formation of filopodia and lamellipodia, AQP3-siRNA inhibited cell invasion and metastasis. Ezrin showed dot redistribution in cells due to suppression of filopodia and lamellipodia (Fig. 7Bb). AQP3 may interact with the ezrin protein, thereby affecting the formation of filopodia and lamellipodia. In the E2P4+siAQP3 group, compared to the E2P4 group, there was reduced distribution of pseudopods, due to AQP3-siRNA inhibiting the formation of pseudopods (Fig. 7Bc). In order to explore the mechanism of AQP3 promoting the migration and invasion, we tested the expression profiles of several molecules related to the EMT, including the epithelial marker E-cadherin and the mesenchymal marker N-cadherin, by western blotting in RL95-2 cells treated with AQP3-siRNA, E2P4, and E2P4+AQP3-siRNA (Fig. 7Ca,b,c,d). N-cadherin protein expression was decreased (Fig. 7Ca,d) (*P* < 0.05), and E-cadherin protein expression was increased (Fig. 7Ca,c) (*P* < 0.05) in the AQP3-siRNA group. N-cadherin expression was increased (Fig. 7Ca,d) (*P* < 0.05) and E-cadherin expression was decreased (Fig. 7Ca,c) (*P* < 0.05) in the E2P4 group. Additionally, AQP3-siRNA inhibited the function of E2 and P4 in the E2P4 and AQP3-siRNA group (*P* < 0.05). The results indicate that AQP3 might act as a positive regulator of the EMT signaling pathways during implantation. These results also suggest that the mechanism underlying EMT promoted by AQP3 results in changes in the shape of cells through altering the actin cytoskeleton and ezrin organization.

## Discussion

Human embryo implantation is a critical complex process involved in embryo apposition and adhesion, followed by penetration and invasion. In order to promote the interaction between the embryo and the uterine endometrium, EECs undergo ultrastructural changes and express multiple molecules during implantation. During the mid-secretory phase of the menstrual cycle (also known as the ‘implantation window’), the EECs need to change their polarity to present an adherent area for the early embryo.

A recently published study using an *in-vitro* model of attachment showed that treatment with ovarian steroid hormones resulted in changes of N-cadherin, E-cadherin and vimentin (mesenchymal cell markers) in Ishikawa cells, accelerated Ishikawa cell motility and increased JAR spheroid outgrowth. This strongly suggested activation of EMT (Uchida *et al.*, 2012; Yu *et al.*, 2016). The conclusion of these authors collectively suggested that the ovarian steroid hormones and the implanting embryo (Jar spheroids mimicking trophoblast) induced EMT of EECs, which may play a key role in early human embryo implantation. Some studies have reported that AQP3 abnormalities contribute to the defects of endometrial receptivity in repeated implantation failure (RIF) patients (Shi *et al.*, 2017) as well as in other species, including mice (Fang *et al.*, 2017) and horses (Klein *et al.*, 2013). In our research, we found that AQP3 expression in the cytoplasm increases during the mid-and late-secretory phases in both luminal and glandular epithelial cells. We also found that AQP3 expression was higher in RL95-2 (high-receptive) cells than in HEC-1A (low-receptive) cells, which are often used to study endometrial receptivity (Dominguez *et al.*, 2005; Liu *et al.*, 2012). Our results indicate that AQP3 may play an important role in endometrial receptivity in humans.

During the menstrual cycle, changes take place in the endometrium to prepare for implantation. The interplay between the gonadal steroids estrogen and progesterone reflects the maturation of the endometrium. Estrogens and progesterone exert their primary action via ligand-specific receptors. Our data showed that treatment with E2 and P4 induced up-regulation of AQP3 expression in RL95-2 cells and HEC-1A cells. As the estrogen concentration increased from 0.1 to 10 μM, the expression of AQP3 increased accordingly, and peaked at 1 μM. Prior research has indicated that a functional ERE is located in the promoter region of the AQP3 gene and may mediate E2-induced up-regulation of AQP3 expression. Our data also showed that P4 up-regulated AQP3 expression by directly targeting the promoter of the AQP3 gene. This is the first report that progesterone exerts its effects by directly targeting AQP3 transcription. E2 and P4 had a more significant effect on AQP3 than did equivalent doses of estrogen or progesterone alone. Hence, we suggest that estrogen and progesterone might have combined effects on AQP3 via ligand-specific receptors.

Ezrin, which is known as a cytoskeletal protein, acts as a link between the plasma membrane and actin cytoskeleton. During cell migration, the transport of adhesion proteins and receptors to filopodia tips has been concerned with their formation and reinforcement (Vasioukhin *et al.*, 2000; Davis *et al.*, 2015), contributing to cell locomotion. Evidence has demonstrated that filopodia can mature into focal adhesions upon lamellipodia advancement (Wong *et al.*, 2014). In the present study, we found that AQP3 and ezrin are binding partners, and AQP3 induces Ezrin reorganization. AQP3 knockdown inhibited the formation of filopodia and lamellipodia and stress fiber rearrangement. An early event in embryo implantation is the disruption of the EEC barrier. The disrupted endometrial epithelial sheet is partially reconstructed by EEC migration and invasion. The embryo is then covered with EEC, and the EEC barrier is restored. Up-regulation of EEC motility is induced by ovarian steroid hormones and implantation-mediated EMT. We found that E2 and P4 up-regulates AQP3 expression to promote human endometrial cells migration and invasion, but not adhesion. We also found that the expression of E-cadherin and N-cadherin proteins changed in relation to the EMT. These results suggest that AQP3 may mediate the EMT by altering cell shape.

Previous studies have indicated that during the implantation process, transforming growth factor-β (TGF-β), epidermal growth factor (EGF) and heparin binding-EGF (HB-EGF) regulate functions of endometrial cells and embryo–maternal interactions (Singh *et al.*, 2011). Studies have demonstrated that growth factors, such as EGF and HB-EGF, up-regulate AQP3 expression through the MAPK/ERK pathway and/or the PI3K/AKT pathway (Ji *et al.*, 2008). In addition, EGF and TGF-β induce EMT through AQP3, thereby altering the shapes of cells (Ryu *et al.*, 2012). Previous studies have indicated that the expression level of AQP3 may have an effect on molecular functions that are important in cell-to-cell and/or cell-to-matrix interactions (Kusayama *et al.*, 2011; Xu *et al.*, 2011). Cell adhesion molecules (intercellular cell adhesion molecule 1 [ICAM-1], integrin α5β1 and E-cadherin) have important effects on embryonic implantation. AQP3 mediates invasion through these cell adhesion molecules which are important EMT markers. In addition, it is well known that intrauterine rings can release copper ions, thus affecting endometrial receptivity. Copper ions inhibit the function of AQP3 (Ji *et al.*, 2008), thus, suggesting that intrauterine rings may affect EEC motility through down-regulation AQP3 expression.

In summary, AQP3 is probably associated with endometrial receptivity and may serve as a marker for the implantation window in vitro. We provide the first report that PR binding to the promoter region of the AQP3 gene mediates regulation of AQP3 expression by P4 in RL95-2 cells. Up-regulation of AQP3 by E2 and P4 in RL95-2 cells increased cell migration and invasion by regulating the expression of EMT-related factors. Increased AQP3 expression may mediate ezrin remodeling and cytoskeleton rearrangement, although other unknown mechanisms may play significant roles during the EMT. Our *in-vitro* research provides pertinent information about the mechanisms of by which AQP3 may affect uterine endometrial receptivity through ovarian steroid hormone-induced motility.

## Authors’ roles

D.C. performed the research and contributed to writing the article. L.S. designed the study and contributed to article revision. X.H. contributed to cell culture. M.Z. contributed to the collection and testing of clinical samples. Z.G., W.C. and X.Y. performed the research. Q.S. and M.D. performed the statistical analyses. T.M. and Y.K. contributed to the study design, data interpretation and article revision. All authors have agreed to be listed and have seen and approved the final article.

## Funding

 National Natural Scientific Grants of China (No. 31570798), the Program for Liaoning Excellent Talents in University (LR2017042), the Doctoral Scientific Research Foundation of Liaoning province (201601236) and the Liaoning Provincial Program for Top Discipline of Basic Medical Sciences.

## Conflict of interest

The authors have declared no conflicts of interest.

## Supplementary Material

Supplementary Data

Supplementary Data
