# Supplementary material for: Aquaporin-3 mediates ovarian steroid hormone-induced motility of endometrial epithelial cells
Source: Hum Reprod. 2018 Oct 4;33(11):2060–73. doi: 10.1093/humrep/dey290 (PMC6195804; doi:10.1093/humrep/dey290)
Supplement: Supplementary Data [file dey290_supplementary_table_si.pdf]

Human Reproduction, 2018
doi:10.1093/humrep/dey290
human reproduction
SUPPLEMENTARY DATA

Supplementary Table SI Location and primer sequence information of promoter region, S1–S6 and S1(D).

Sequence name	Start site in the transcript	end site in the transcript	chr9 start	chr9 end	Sequence information	Primer information	
Promotor region	+50	−2000	33447560	33449609	CTGGGCAACATAGCAAGACCTCATTTTTACAACAGATTTTTAAAAATTAGCTGAGTGTCATGGTGGCACATAGT CCCTATAGTCCCATCCATTCTGGA<br>GGCTGAGGTGGGAGGATCACTTGGGCCCAGGAGTTCGAGGCTACAGTGAGCTGTGATTGCACCACAGCCTGGGTGATGGAGCAAGACCCTA<br>CTCTTTAAAAAGAGAGAGAGGGCAAGGTTGTCCCATGATCCCCCTCAGATAGGGCTGACTCCCCCATAATTAAGTTTGCTTAAAGAGGCAGA<br>CTTGCTGGGCTCAGTGGCTCACGCCTGTAATCCCAGCACTTTGGGAGGCTGAGGCGGGTGGATAACTTGTGGTCAGGAGTTCGAAATCAGCCTGG<br>TCAACATGGTGAATCCCCATCTCCACTAAAAAAACAAAAATTAGCTGGGCATGGTGGTGTGCACCTGTAATCCCAGCT AATCGGGAGGCTGAAGCAGG<br>AGAATCACTTGAACCTGGGAGGTGGAGGTTGCAGGGAGCCGAGATCACGCCATTGCACTCCACCTAGGCGACAGAGAGAGACTCCGTC<br>TAAAAAAAAAAAAGAGACAGACTCTTCCTTGGCTGGGGGTAAGTCAGATGGGAGAGGAGAGGGTTAAAAACAGCTGGGACTCAGCCTGCTGGCAAAC<br>ATGTGGCATGTGGCATGTCGGGGCAACTGCAGCTCAGCCTCTGGAGCCATGTGAGCAATGCACGCAGGTACACGTGTGACAAGCTAGGTCACCTAG<br>CCATGTTCAACAGGCATGTGCACAGCCACGAGGAATGCCCAGCCGTACAATTAGGCACACAGGACATCCGCCATGTGTAGACACAGCTGTGGAC<br>ATAGCTGGCCAGGACATGCGACACACGACGTGCTCATAGCACAGGGAGAAGGGCCCATGAAGTCTGGTTGGAACTCAGCACGTG<br>TGTCTGTGTGCCCACCTGAGTCTGGACTGCTGCCCCTCTGACACTAGCTGTCCCCTTGAAGGGTCGGTGCCTTATCTGTCCTGACAGAAGAGACA<br>GTGTTGCTTCTCACTTGGGGCTCGCAGCCTCCTCCTCCTGCCTCGAACTGAGGATCTGTTGGGTCCAGTCATCCTGGAGAGATGCGGCC<br>AGTTTCTTTCTGACAGGTCTCCTCCTGCCCGCAAGGAAGTGGGGTGATCACAGGGCGCAGGTGGTCTCTATGACAGCTGCATCCTCTCCAGCCATGG<br>CCCTGAACCCTGCCTATAATCCCACCATTGGCTCTCAGATCTGCCTAAGCCTCTCAGCCCCCTTGACGTCCCCTCCCTTAAGCGCCCTCC<br>GAAGGCCACCCGTCCCTCAAAGCTCCTCACACTCCATGCCCGCAGCTCCCTCCACCCGGCGTCCGCACCAGCCTCCCAGCCGAGGT<br>GGGGCGGGGGCGAGGGGGCGCGCACTCCTCGGCGCTCCGGGACTGCAGGGCG GGGCTGCAGGGCGGGCGGGGCCCGTGTCTCCAGCG<br>CTCCTATAAAGGGAGCCACCAGCGCTGGAGGCCGCTGCTCGCTGCGCCACCGCCTCCCGCCACCCCTG		
S1	+22	−245	33447588	33447854	CTCGTCGCCGGAGGTCGCGACCACCGAGGGAAATATCCTCGCGACCTCTGTGCCCG<br>GGG CGGGCGGGACGTCGGGGCGGGACGTCAGGGCCTCGCGGCTCCTCACGCGCGG<br>GGGAGCGGGGGCGGGGTGGAGCCGACCCTCCGACCACGCCTGCGGCCCACCTCCC<br>TCGACGCCCGTACCTCACACTCCTCGAAACTCCCTGCCCACCGGAAGCCTCCCGCGA<br>ATTCCCTCCCCTGCAGTTCCCCCGACTCTCCGAATCCGTCTAGA	Forward primer<br>Reverse primer	TCCATGCCCGCAGCTCCCTCCA<br>CTCCAGCGCTGGTGGCTCCCTTTA
S2	−442	−615	33448054	33448226	CGCTCGGGGTTCACTCTTCGTTGTGACAGAGAAGACAGTCCTGTCTATTCCGTGGCT<br>GGGAAGTTCCCCTGTCGATCACAGTCTCCCCGTCGTCAGGTCTGAGTCCACCCGTGT<br>GTCTGTGTGCACGACTCAAGGTTGGTCTGAAGTACCCGGGAAGAGGGACACGATACTCG	Forward primer<br>Reverse primer	CCAGGACATGCGACACACGA<br>AGCCCCAAGTGAGAAGCAAC
S3	−1211	−1416	33448382	33448503	ACGCACGTAACGAGTGTACCGAGGTCTCCGACTCGACGTCAACGGGGCTGTACGGTG<br>TACGGTGTACAAACGGTCGTCCGACTCAGGGTCGACAAAAATTGGGAGAGGAGAGGG<br>TAGACTGA	Forward primer<br>Reverse primer	AGACTCTTCCTTGGCTGGG<br>ACCTGCGTGCATTGCTCAC
S4	−1214	−1439	33448825	33449050	CAGACGGAGAAATTCGTTTGAATTAATACCCCCTCAGTCGGGATAGACTCCCCCTAGTAC<br>CCTGTTGGAACGGGAGAGAGAGAAAAATTTCTCATCCCAGAACGAGGTAGTGGGTCCGA<br>CACCACGTTAGTGTCGAGTGACATCGGAGCTTGAGGACCCGGGTTCACTAGGAGGGT<br>GGAGTCGGAGGTCTTACCTACCCTGATATCCCTGATACACGGTGGTACTG	Forward primer<br>Reverse primer	GTCATGGTGGCACATAGTC<br>GTCTGCCTCTTTAAGCAAACT
S5	−1426	−1599	33449037	33449210	ACACGGTGGTACTGTGAGTCGATTAAAAATTTTTAGACAACATTTTTACTCCAGAACGAT<br>ACAACGGGTCCAATCAGAGTTTGAGGACCGAAATTTGCTAGGAGGGTGGAGCCGGAGG<br>GCTTCACGTTGGAACGGGAGATACGACGAAAATTATTGTTCCTTTGTAGACCACCG	Forward primer<br>Reverse primer	GCCACCAGATGTTTCCTTG<br>TGTGCCACCATGACACTCA
S6	−1569	−1690	33449208	33449301	CCGGACCCTCGTTTGTTGTACGGGAATCCGACTCGAAGATACCGTCGTAACGGTCT<br>CCTCTAGTTTCTTTAGTAAAAGGTCTCCCTGAATTAGG	Forward primer<br>Reverse primer	TCCTCAGGGTCATTAGCCCA<br>CTGGGAGCAAACAACATGCC
S1 (D)	‘CTGCAGGGCGGGCGGGGC CCGTGTCTCCAGCGCTCC TATAAAGGGAGCCA’ was deleted in S1				CTCGTCGCCGGAGGTCGCGACCGGGGCGGGACGTCAGGGCCTCGCGGCTCCTCACGCG<br>CGGGGGAGCGGGGGCGGGGTGGAGCCGACCCTCCGACCACGCCTGCGGCCCACCTCCC<br>TCGACGCCCGTACCTCACACTCCTCGAAACTCCCTGCCCACCGGAAGCCTCCCGCGAATTCCCT<br>CCCCTGCAGTTCCCCCGACTCTCCGAATCCGTCTAGA	supply by Gene Pharma company, Shanghai,China	
