# Supplementary material for: Aquaporin-3 mediates ovarian steroid hormone-induced motility of endometrial epithelial cells
Source: Hum Reprod. 2018 Oct 4;33(11):2060–73. doi: 10.1093/humrep/dey290 (PMC6195804; doi:10.1093/humrep/dey290)
Supplement: Supplementary Data [file dey290_supplementary_table_sii.pdf]

Supplementary Table SII **Antibody information.**

Antibody	Company	Catalog number	Species reactivity	Source	WB concentration (µg/ml)	IHC concentration (µg/ml)	IF concentration (µg/ml)	IP concentration (µg/ml)
Anti-aquaporin 3	Abcam	ab125219	Mouse, rat, human	Rabbit	1	5	5	
Anti-aquaporin 3	Snata			Goat	1	5	5	
Ezrin antibody	Cell Signaling Technology	#3145	Human, mouse, rat, monkey, bovine	Rabbit	1		10	20
E-cadherin antibody	Proteintech	20874-1-AP	Human, mouse, rat	Rabbit	1			
N-cadherin antibody	Proteintech	22018-1-AP	Human, mouse, rat, Gekko japonicus	Rabbit	1			
F-actin antibody	Abcam	ab205	Mouse, rat, rabbit, human	Mouse	1			
β-Tubulin	Proteintech	10094-1-AP	Human, mouse, rat, hamsters	Rabbit	1			
